# Identification of a human peripheral blood monocyte subset that differentiates into osteoclasts

**DOI:** 10.1186/ar2046

**Published:** 2006-09-21

**Authors:** Yukiko Komano, Toshihiro Nanki, Kenji Hayashida, Ken Taniguchi, Nobuyuki Miyasaka

**Affiliations:** 1 Department of Medicine and Rheumatology, Graduate School, Tokyo Medical and Dental University, Tokyo 113-8519, Japan; 2 The 21st Century Center of Excellence Program for the Frontier Research on Molecular Destruction and Reconstruction of Tooth and Bone, Tokyo Medical and Dental University, Tokyo 113-8519, Japan; 3 Department of Orthopedic Surgery, Hoshigaoka Koseinenkin Hospital, Osaka 573-8511, Japan; 4 Division of Rheumatic Diseases, Tokyo Metropolitan Bokutoh Hospital, Tokyo 130-0022, Japan

## Abstract

Increased bone resorption mediated by osteoclasts causes various diseases such as osteoporosis and bone erosion in rheumatoid arthritis (RA). Osteoclasts are derived from the monocyte/macrophage lineage, but the precise origin remains unclear. In the present study, we show that the purified CD16^- ^human peripheral blood monocyte subset, but not the CD16^+ ^monocyte subset, differentiates into osteoclast by stimulation with receptor activator of NF-κB ligand (RANKL) in combination with macrophage colony-stimulating factor (M-CSF). Integrin-β3 mRNA and the integrin-αvβ3 heterodimer were only expressed on CD16^- ^monocytes, when they were stimulated with RANKL + M-CSF. Downregulation of β3-subunit expression by small interfering RNA targeting β3 abrogated osteoclastogenesis from the CD16^- ^monocyte subset. In contrast, the CD16^+ ^monocyte subset expressed larger amounts of tumor necrosis factor alpha and IL-6 than the CD16^- ^subset, which was further enhanced by RANKL stimulation. Examination of RA synovial tissue showed accumulation of both CD16^+ ^and CD16^- ^macrophages. Our results suggest that peripheral blood monocytes consist of two functionally heterogeneous subsets with distinct responses to RANKL. Osteoclasts seem to originate from CD16^- ^monocytes, and integrin β3 is necessary for osteoclastogenesis. Blockade of accumulation and activation of CD16^- ^monocytes could therefore be a beneficial approach as an anti-bone resorptive therapy, especially for RA.

## Introduction

Rheumatoid arthritis (RA) is an autoimmune disease characterized by chronic inflammation and proliferation of the synovium in multiple joints. A large number of inflammatory cells, including T cells, B cells, macrophages and dendritic cells, accumulate in the affected synovium, and these inflammatory cells, together with fibroblast-like synoviocytes, express various cytokines, such as tumor necrosis factor alpha (TNFα), IL-6 and receptor activator of NF-κB ligand (RANKL), which are known to induce differentiation and activation of osteoclasts. The inflammatory synovial tissue, known as pannus, invades the articular bone and causes focal bone erosion, which is the hallmark of RA. Histopathologically, osteoclasts are present at the interface of the pannus and bone. Interestingly, the deletion of RANKL or c-*Fos *gene, which is important for osteoclastogenesis, results in minimal bone destruction in mouse models of arthritis [1,2]. Furthermore, other studies indicated that inhibition of osteoclastogenesis by osteoprotegerin, a decoy receptor for RANKL, limits bone destruction in experimental models of arthritis. These studies suggest that osteoclasts are involved in focal bone erosion in RA [3].

Osteoclasts are derived from the monocyte/macrophage lineage. It is reported that osteoclast precursors reside in human peripheral blood monocytes [4,5]. A marked increase of the circulating osteoclast precursors was demonstrated in patients with erosive psoriatic arthritis as well as in arthritic TNFα transgenic mice [6,7]. It was also shown that peripheral monocytes differentiate into osteoclasts when seeded on RANKL/osteoclast differentiation factor-producing RA synovial fibroblasts [8]. In addition, RA synovial macrophages thought to originate from peripheral blood monocytes were shown to differentiate into osteoclasts [9,10]. Monocytes are therefore involved not only in synovial inflammation, but also in bone remodeling as potential precursors for synovial macrophages and osteoclasts.

Human peripheral blood monocytes consist of two major subsets, CD16^+ ^and CD16^-^, comprising 5–10% and 90–95% of the monocytes, respectively. These two subsets exhibit different chemotaxis activities and potential of cytokine production [11,12]. Moreover, activation of the Toll-like receptor induces distinct subsets, CD1b^+ ^dendritic cells and DC-SIGN^+ ^(dendritic cell-specific C-type lectin ICAM-3-grabbing nonintegrin) macrophages from CD16^+ ^and CD16^- ^monocytes, respectively [13]. It has not been revealed, however, which monocyte subset develops into osteoclasts.

In the present study, we determined the human peripheral blood monocyte subset that differentiates into osteoclasts, and revealed that each subset exhibits a different response for osteoclastogenic stimuli.

## Materials and methods

### Purification of peripheral blood monocytes

Peripheral blood monocytes from healthy donors were collected using Ficoll-Conray (Imuuno-Biological Laboratories, Gunma, Japan) gradient centrifugation. Negative selection of monocytes was performed using MACS microbeads (Miltenyi Biotec, Auburn, CA, USA) according to the protocol supplied by the manufacturer.

The purified monocytes were separated into two subsets, CD16^+ ^and CD16^- ^monocytes, using CD16 MicroBeads (Miltenyi Biotec). Flow cytometry analysis using FITC-conjugated mouse anti-CD14 mAb (MY4; Bechman Coulter, Fullerton, CA, USA) and phycoerythin-conjugated mouse anti-CD16 mAb (3G8; BD Biosciences, San Jose, CA, USA) showed that the purities of the CD16^+ ^and CD16^- ^monocytes were more than 90% and 92%, respectively.

For the other experiment, monocytes were purified using CD14 MicroBeads (Miltenyi Biotec), and then stained either with FITC-conjugated mouse anti-CD33 mAb (MY9; Bechman Coulter) or phycoerythin-conjugated mouse anti-CD16 mAb (3G8). Cell sorting of the stained cells was performed using a FACS Vantage cytometer (BD Biosciences) or a MoFlo cell sorter (Dako, Glostrup, Denmark).

### Osteoclast differentiation

Purified CD16^+ ^and CD16^- ^monocytes (5 × 10^4 ^cells/well) were incubated in 96-well plates in αMEM (Sigma, St Louis, MO, USA) with heat-inactivated 10% fetal bovine serum (FBS) (Sigma) or with Ultra-Low IgG FBS (IgG < 5 μg/ml; Invitrogen, Carlsbad, CA, USA), and where indicated with M-CSF + RANKL (Peprotech, Rocky Hill, NJ, USA).

For the other experiments, varied numbers of CD16^+ ^monocytes (1 × 10^3^, 2.5 × 10^3^, 5 × 10^3^) were mixed with CD16^- ^monocytes (5 × 10^4 ^cells/well), and were cultured in 96-well plates in αMEM with heat-inactivated 10% FBS. The medium was replaced with fresh medium 3 days later, and after incubation for 7 days the cells were stained for tartrate-resistant acid phosphatase (TRAP) expression using a commercial kit (Hokudo, Sapporo, Japan). The number of TRAP-positive multinucleated cells (MNC) in three randomly selected fields examined at 100× magnification or the total number of TRAP-positive MNC per well was counted under light microscopy.

### Resorption assay

Monocytes were seeded onto plates coated with calcium phosphate thin films (Biocoat Osteologic; BD Biosciences) and were incubated with RANKL (40 ng/ml) + M-CSF (25 ng/ml) for 7 days. The cells were then lysed in bleach solution (6% NaOCl, 5.2% NaCl). The resorption lacunae were examined under light microscopy.

### Enzyme-linked immunosorbent assay

Purified monocytes were cultured in 96-well plates where indicated either with RANKL or M-CSF for 24 hours. Concentrations of TNFα and IL-6 in the culture supernatant were measured with an ELISA kit (BioSourse International, Camarillo, CA, USA). For experiments of matrix metalloproteinase (MMP)-9 and TRAP-5b, culture supernatants were collected on day 7 and the concentrations of these enzymes were measured using an MMP-9 ELISA kit (Amersham Biosciences, Piscataway, NJ, USA) or a TRAP-5b ELISA kit (Suomen, Turku, Finland).

### Reverse transcriptase-polymerase chain reaction

Monocytes (1 × 10^6 ^cells/well) were cultured in six-well plates with M-CSF alone or with M-CSF + RANKL for 3 days. Total RNA was extracted using RNeasy Micro (Qiagen, Valencia, CA, USA). The RNA was then treated with DNase I (Qiagen). The oligo(dT)-primed cDNA was synthesized using Superscript II reverse transcriptase (Invitrogen). The amount of cDNA for amplification was adjusted by the amount of RNA measured by an optical density meter and also by β-actin or GAPDH PCR products. One microliter of cDNA was amplified in a 50 μl final volume containing 25 pmol appropriate primer pair, 10 pmol each of the four deoxynucleotide triphosphates, and 5 units FastStart Taq DNA Polymerase (Roche, Manheim, Germany) in a thermal cycler (PTC-200; MJ GeneWorks, Waltham, MA, USA).

The PCR conditions were 25–40 cycles of denaturation (95°C for 30 s), annealing (60–62°C for 1 min) and extension (72°C for 1 min). The sequences of the primers are presented in Table 1. The PCR products were separated by electrophoresis through 2% agarose gel.

### Western immunoblot analysis

Purified monocytes were cultured for 3 days in the presence of 40 ng/ml M-CSF with or without 25 ng/ml RANKL. Cells were lysed in RIPA Lysis buffer (upstate, Lake Placid, NY, USA) containing protease inhibitors (Roche) for 15 min at 4°C. A total of 20 μg protein was boiled in the presence of 6 × sodium dodecyl sulfate sample buffer, and was separated on 7.5% or 10% sodium dodecyl sulfate-polyacrylamide gel (ATTO, Tokyo, Japan). Proteins were then electrotransferred to a polyvinylidene fluoride microporous membrane (Millipore, Billerica, MA, USA) in a semidry system. Membranes were incubated in 10% skim milk prepared in phosphate-buffered saline (PBS) containing 0.1% Tween 20, and were subjected to immunoblotting. Antibodies used were goat anti-RANK antibody (Techne Corporation, Minneapolis, MN, USA), goat anti-c-fms antibody (R&D systems, Minneapolis, MN, USA), and mouse anti-β-actin mAb (AC-15; Sigma). Peroxidase-conjugated rabbit anti-goat IgG antibody (Dako) or peroxidase-conjugated rabbit anti-mouse IgG antibody (Dako) was used as the second antibody. The signals were visualized by chemiluminescence reagent (ECL; Amersham Biocsiences, Little Chalfont, UK).

### Cell surface expression of c-fms

The following mAbs were used for analysis of c-fms expression: Alexa 647-conjugated anti-CD14 mAb (UCHM1; Serotec, Oxford, UK), FITC-conjugated anti-CD16 mAb (3G8; Bechman Coulter) and phycoerythin-conjugated anti-c-fms mAb (61708; R&D systems). Alexa 647-conjugated mouse IgG2a (Serotec), FITC-conjugated mouse IgG_1 _(BD Biosciences) and phycoerythin-conjugated mouse IgG_1 _(Bechman Coulter) were used as isotype controls. Peripheral blood monocytes (1 × 10^5 ^cells) were incubated with 1 μg human IgG for 15 minutes, and were then stained with three fluorochrome-labeled mAbs for 45 minutes on ice. The stained cells were analyzed with a FACS Calibur (BD Biosciences).

### Immunofluorescent staining

Monocytes (8 × 10^4 ^cells/well) were allowed to adhere on 96-well plates overnight or were cultured with M-CSF and RANKL for 2–4 days. The cells were fixed in acetone and then stained with anti-αvβ3 mAb (LM609; Chemicon, Temecula, CA, USA) or mouse IgG_1 _(11711; R&D Systems) as an isotype-matched control. Alexa fluor546-conjugated goat anti-mouse IgG_1 _antibody (Molecular Probes, Eugene, OR, USA) was used as the second antibody. TOTO-3 (Molecular Probes) was used for nuclear staining.

### Flow cytometric analysis of p38 MAPK and ERK1/2 phosphorylation

Purified monocytes were cultured in the presence of 25 ng/ml M-CSF for 3 days, and were either left unstimulated or were stimulated with 40 ng/ml RANKL at 37°C. Stimulations were stopped by adding an equal volume of PhosFlow Fix Buffer I solution (BD Biosciences) to the cell culture. After incubation for 10 minutes at 37°C, the cells were permeabilized by washing twice at room temperature in PhosFlow Perm/Wash Buffer I (BD Biosciences). A total of 1 × 10^5 ^cells was then Fc blocked with 1 μg human IgG for 15 minutes, and was stained with Alexa Fluor 647-conjugated mAb either to phospho-p38 MAPK (T180/Y182) or to phospho-ERK1/2 (T202/Y204) (BD Biosciences) for 30 minutes at room temperature. Alexa Fluor 647-conjugated mouse IgG_1 _(BD Biosciences) was used as an isotype control. The cells were washed in PhosFlow Perm/Wash Buffer I, and were analyzed by flow cytometry, as already described.

### RNA interference

RNA oligonucleotides (iGENE, Tsukuba, Japan) were designed based on the algorithm that incorporates single nucleotide polymorphism and homology screening to ensure a target-specific RNA interference effect. The following sense and antisense oligonucleotides were used: integrin β3, 5'-GCU UCA AUG AGG AAG UGA AGA AGC A-AG and 3'-UA-CGA AGU UAC UCC UUC ACU UCU UCG U; randomized control, 5'-CGA UUC GCU AGA CCG GCU UCA UUG C-AG and 3'-UA-GCU AAG CGA UCU GGC CGA AGU AAC G; and lamin, 5'-GAG GAA CUG GAC UUC CAG AAG AAC A-AG and 3'-UA-CUC CUU GAC CUG AAG GUC UUC UUG U.

CD16^- ^monocytes (8 × 10^4 ^cells/well) were incubated in 96-well plates in optimem (Invitrogen). After 1 hour, siRNAs were transfected into the cells using oligofectamine (Qiagen) based on the method recommended by the manufacturer. After 2 hours, the cells were washed once with PBS, followed by the addition of αMEM supplemented with 10% FBS, M-CSF and RANKL. After a 2-day incubation, the β3 mRNA expression was analyzed by RT-PCR with different PCR cycles, as described earlier. Immunofluorescent staining for the αvβ3 heterodimer was also performed as described above, and numbers of αvβ3-positive cells were counted in randomly selected three fields at 100× magnification. Seven days after the transfection of siRNAs, the number of TRAP-positive MNC in five fields examined at 100× magnification was counted under light microscopy.

### Inhibition of osteoclastogenesis with cyclic RGDfV peptide

CD16^- ^monocytes were incubated in 96-well plates with M-CSF + RANKL for 2 days. A medium containing either cyclic RGDfV peptide (Arg-Gly-Asp-D-Phe-Val) (Calbiochem, San Diego, CA, USA) or dimethyl sulfoxide was then added. After incubation for a further 5 days, the number of TRAP-positive MNC in five fields examined at 100× magnification was counted under light microscopy.

### Immunohistochemistry

Synovial tissue samples were obtained during total knee joint replacement surgery from four RA patients. Signed consent forms were obtained before the operation. The experimental protocol was approved by the ethics committee of the Tokyo Medical and Dental University. RA was diagnosed according to the American College of Rheumatology criteria [14].

Double immunofluorescent staining for CD68 and CD16 antigens was conducted on optimal cutting temperature-embedded sections of frozen synovial samples. Eight-micrometer-thick cryostat sections of RA synovium were fixed in acetone for 3 minutes and were then rehydrated in PBS for 5 minutes. The samples were incubated in 5 μg/ml proteinase K (Roche), 50 mM ethylenediamine tetraacetic acid, 100 mM Tris–HCl, pH 8.0, for 15 minutes at room temperature followed by a wash in PBS. The samples were then blocked with 10% goat serum in PBS for 60 minutes at room temperature, and were incubated with anti-CD16 mAb (3G8; Immunotech, Marseille, France) or mouse IgG_1 _(11711) as an isotype-matched control in 1% bovine serum albumin/PBS for 60 minutes at room temperature. The samples were then washed three times in PBS, for 5 minutes each, and incubated with Alexa fluor546-conjugated goat anti-mouse IgG_1 _antibody (Molecular Probes) in 1% bovine serum albumin/PBS for 60 minutes at room temperature. The samples were then sequentially stained for CD68 antigen in a manner similar to that used for CD16 staining. The samples were stained with anti-CD68 mAb (PGM1; Immunotech) or mouse IgG_3 _(6A3; MBL, Nagoya, Japan) followed by labeling with Alexa fluor488-conjugated goat anti-mouse IgG_3 _antibody (Molecular Probes). The samples were examined by confocal laser scanning microscope (Olympus, Tokyo, Japan).

### Statistical analysis

Data are expressed as the mean ± standard error of the mean. A nonpaired Student's *t *test was used for comparison, using the StatView program (Abacus Concepts, Berkeley, CA, USA). *P *< 0.05 was considered statistically significant.

## Results

### Induction of osteoclasts from CD16^- ^peripheral blood monocytes

**Figure 1:**
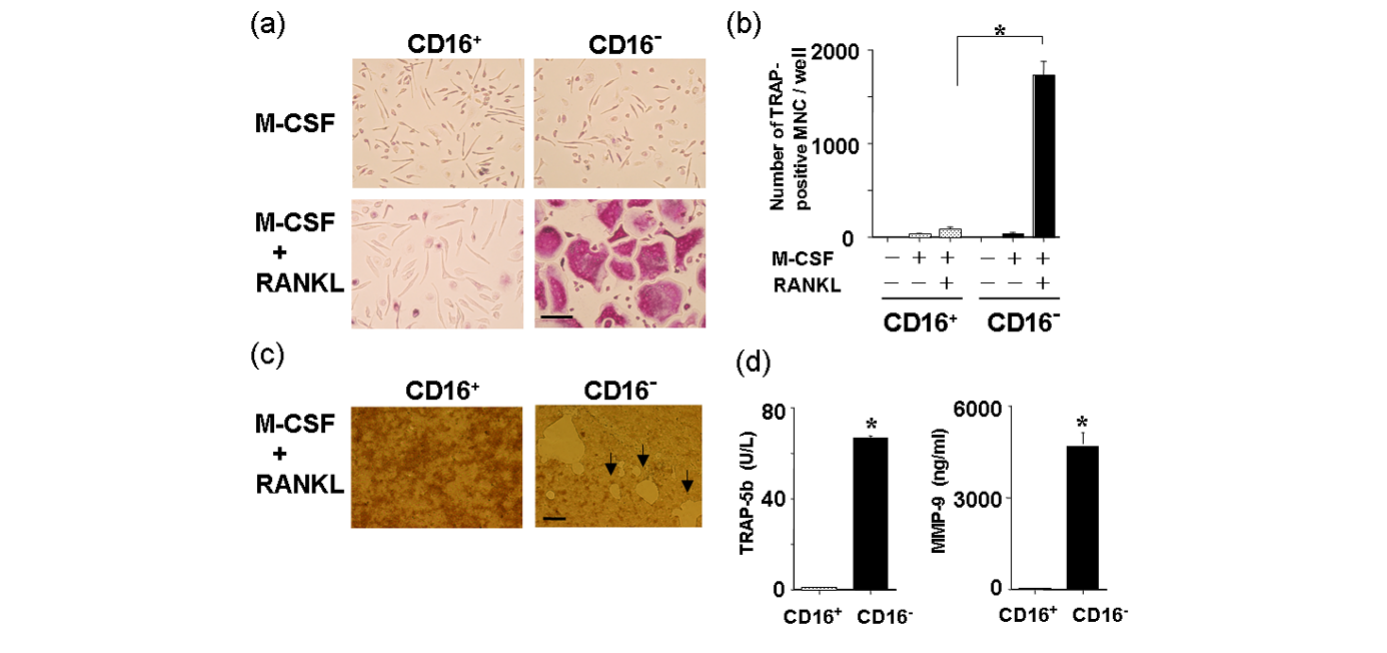
Induction of osteoclasts from human peripheral blood monocytes. (a) Purified CD16^+ ^and CD16^- ^peripheral blood monocytes were cultured with either macrophage colony-stimulating factor (M-CSF) (25 ng/ml) alone or with M-CSF (25 ng/ml) + receptor activator of NF-κB ligand (RANKL) (40 ng/ml) for 7 days and were stained for tartrate-resistant acid phosphatase (TRAP) activity. Original magnification, ×100. (b) The number of TRAP-positive multinucleated cells (MNC) (three or more nuclei) that differentiated from each monocyte subset was counted. (c) Resorbtive activity was assessed by culturing monocytes on plates coated with calcium phosphate films. The cells were treated with M-CSF (25 ng/ml) and RANKL (40 ng/ml) for 7 days. Arrows show resorbed lacunae. Original magnification, ×100. (d) Culture supernatants of CD16^+ ^and CD16^- ^were collected on day 7, and the concentrations of TRAP-5b and MMP-9 were measured with an ELISA. Representative data of more than three independent experiments are shown. Data represent the mean ± standard error of the mean values of duplicate or triplicate wells. *P < 0.01. Scale bars = 100 μm.

To identify the monocyte subset that differentiates into osteoclasts, we examined osteoclast formation from CD16^+ ^and CD16^- ^human peripheral blood monocytes. The monocyte subsets were purified using magnetic beads. Incubation with M-CSF alone did not induce osteoclast formation from either subset (Figure 1a). Culture with M-CSF + RANKL induced a significant number of TRAP-positive MNC from the CD16^- ^subset, whereas only few CD16^+ ^monocytes differentiated into TRAP-positive MNC (Figure 1a,b). We then assessed the bone resorptive ability by culturing cells on calcium phosphate-coated plates with M-CSF + RANKL. Resorption lacunae were detected only in the CD16^- ^subset (Figure 1c), indicating the TRAP-positive CD16^-^-derived MNC possessed the osteoclast phenotype.

Similar results were obtained using purified monocytes by FACS sorting (purities: CD16^+^, 96%; CD16^-^, 97%). The number of TRAP-positive MNC induced were 36 ± 3 cells/well and 348 ± 13 cells/well from CD16^+ ^and CD16^- ^monocytes, respectively. To exclude the possibility that the anti-CD16 antibody used for isolation of CD16^+ ^monocytes inhibits osteoclast formation, we separated the two subsets, CD33^low ^monocytes and CD33^high ^monocytes, using anti-CD33 mAb and a fluorescent cell sorter, since it was reported that CD33^low ^monocytes correspond to CD16^+^, and that CD33^high ^correspond to CD16^- ^monocytes [15]. On average, the CD33^low ^population contained CD16^- ^(10.2%)/CD16^+ ^(89.8%) monocytes, and the CD33^high ^population contained CD16^- ^(86.3%)/CD16^+ ^(13.7%) monocytes.

Culture with M-CSF + RANKL induced TRAP-positive MNC from CD33^high ^monocytes, whereas no or few CD33^low ^monocytes differentiated into TRAP-positive MNC (CD33^low ^vs CD33^high^, 2 ± 1 vs 192 ± 71 cells/well; *n *= 3). TRAP-5b and MMP-9 in the culture supernatants, both of which are known to be produced by osteoclasts, were measured by ELISA. The concentrations of both enzymes were significantly higher in the culture supernatant of CD16^- ^monocytes than in that of CD16^+ ^monocytes (Figure 1d). These results suggest that the CD16^- ^peripheral blood monocyte subset, but not the CD16^+ ^subset, differentiate into osteoclasts by incubation with RANKL + M-CSF.

### CD16^+ ^monocytes do not affect the osteoclastogenesis from CD16^- ^monocytes

**Figure 2:**
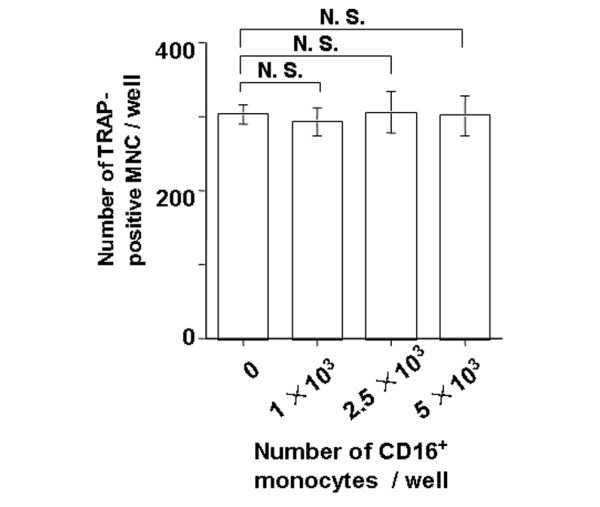
Effect of CD16^+ ^monocytes on the osteoclastogenesis from CD16^- ^monocytes. CD16^+^monocytes (0, 1 × 10^3^, 2.5 × 10^3^, 5 × 10^3 ^cells/well) were mixed with CD16^- ^monocytes (5 × 10^4 ^cells/well) in 96-well plates, and were cultured for 7 days in the presence of macrophage colony-stimulating factor (M-CSF) + receptor activator of NF-κB ligand (RANKL). The number of tartrate-resistant acid phosphatase (TRAP)-positive multinucleated cells (MNC) induced was counted. Representative data of two independent experiments are shown. Data represent the mean ± standard error of the mean values of quadriplicate wells. N.S., not significant.

To examine whether CD16^+ ^monocytes affect osteoclastogenesis from CD16^- ^monocytes, varied numbers of CD16^+ ^monocytes were mixed with CD16^- ^monocytes (5 × 10^4 ^cells/well), and were cultured for 7 days in the presence of M-CSF + RANKL. The number of TRAP-positive MNC was not altered by the presence of CD16^+ ^monocytes (Figure 2). The results indicated that CD16^+ ^monocytes did not hamper or enhance the osteoclastogenesis from CD16^- ^monocytes.

### Differences in cytokine production by RANKL-stimulated or M-CSF-stimulated CD16^+ ^and CD16^- ^monocytes

**Figure 3:**
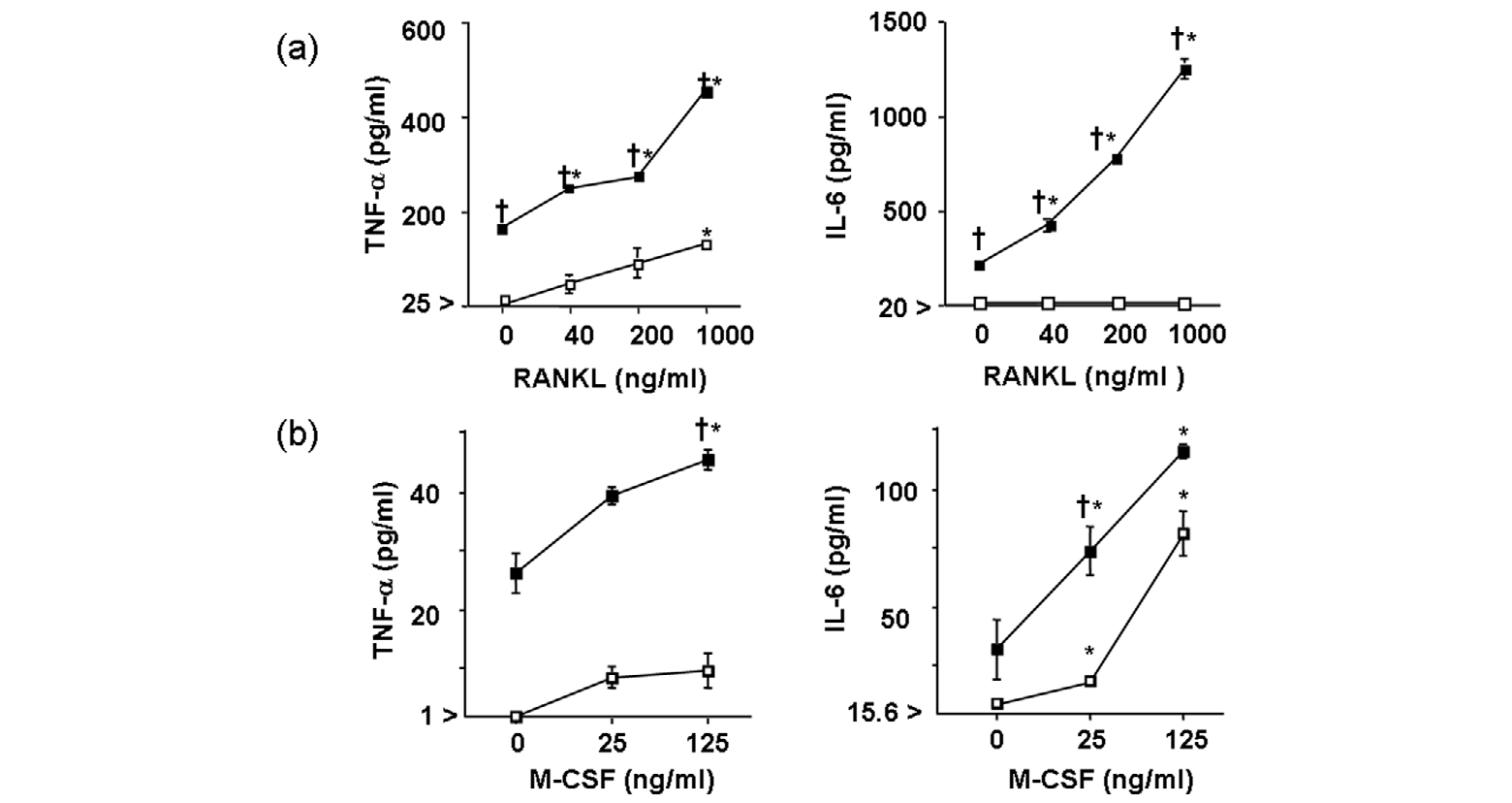
Tumor necrosis factor α and IL-6 production by monocyte subsets with stimulation. Purified CD16^+ ^and CD16^- ^peripheral blood monocytes were incubated either with receptor activator of NF-κB ligand (RANKL) (0, 40, 200, 1000 ng/ml) or macrophage colony-stimulating factor (M-CSF) (0, 25, 125 ng/ml) for 24 hours. Tumor necrosis factor alpha (TNFα) and IL-6 concentrations in the culture supernatant were measured by ELISA. Results are representative of more than three independent experiments. Open squares, CD16^+ ^monocytes; filled squares, CD16^+ ^monocytes. Data are the mean ± standard error of the mean values of duplicate wells. *P < 0.03, no stimulation vs either RANKL or M-CSF stimulation; ^†^*P *< 0.03, CD16^+ ^vs the CD16^- ^monocyte subset.

To compare the biological response of CD16^+ ^and CD16^- ^subsets with either RANKL or M-CSF stimulation, we measured the amount of TNFα and IL-6 production after exposure of cells to various concentrations of RANKL or M-CSF with an ELISA. Without RANKL the CD16^+ ^subset produced a significant amount of TNFα and IL-6, whereas the CD16^- ^subset produced undetectable levels (Figure 3a). RANKL stimulation increased TNFα production from both subsets in a dose-dependent manner, although the CD16^+ ^subset produced more TNFα than did the CD16^- ^subset. RANKL stimulation also enhanced IL-6 production from the CD16^+ ^subset, but not from the CD16^- ^subset. M-CSF stimulation increased TNFα and IL-6 production from both subsets, although the CD16^+ ^subset produced more than the CD16^- ^subset (Figure 3b).

These results suggest that CD16^+ ^monocytes also respond both to RANKL and M-CSF stimulation, although such stimulation does not result in differentiation into osteoclasts. CD16^+ ^monocytes were also noted to express higher amounts of inflammatory cytokines compared with CD16^- ^monocytes with or without RANKL or M-CSF stimulation.

### Comparison of expression levels of molecules involved in osteoclastogenesis between CD16^+ ^and CD16^- ^monocytes

Diverse molecules are involved in RANKL/RANK and its costimulatory signal transduction pathways [16]. The different response to RANKL + M-CSF stimulation between the CD16^+ ^monocyte subset and the CD16^- ^monocytes subset might be explained by the expression profiles of these molecules. We therefore examined the mRNA levels of the following molecules: receptor activator of NF-κB (RANK), the receptor for RANKL; c-fms, the receptor for M-CSF; tumor necrosis factor receptor-associated factor 6 (TRAF-6), the adaptor protein for RANK; c-Fos and nuclear factor of activated T cells c1 (NFATc1), transcription factors that are essential for osteoclastogenesis; DNAX-activation protein 12 (DAP12) and Fc receptor γ chain (FcRγ), adaptor proteins known to deliver costimulatory signals in RANKL-induced osteoclastogenesis; signal regulatory protein β1 (SIRP-β1), triggering receptor expressed on myeloid cells 2 (TREM-2) and osteoclast-associated receptor (OSCAR), transmembrane receptors that associate with either DAP12 or FcRγ; and αv and β3, integrins known to be expressed as the αvβ3 heterodimer on osteoclasts.

**Figure 4:**
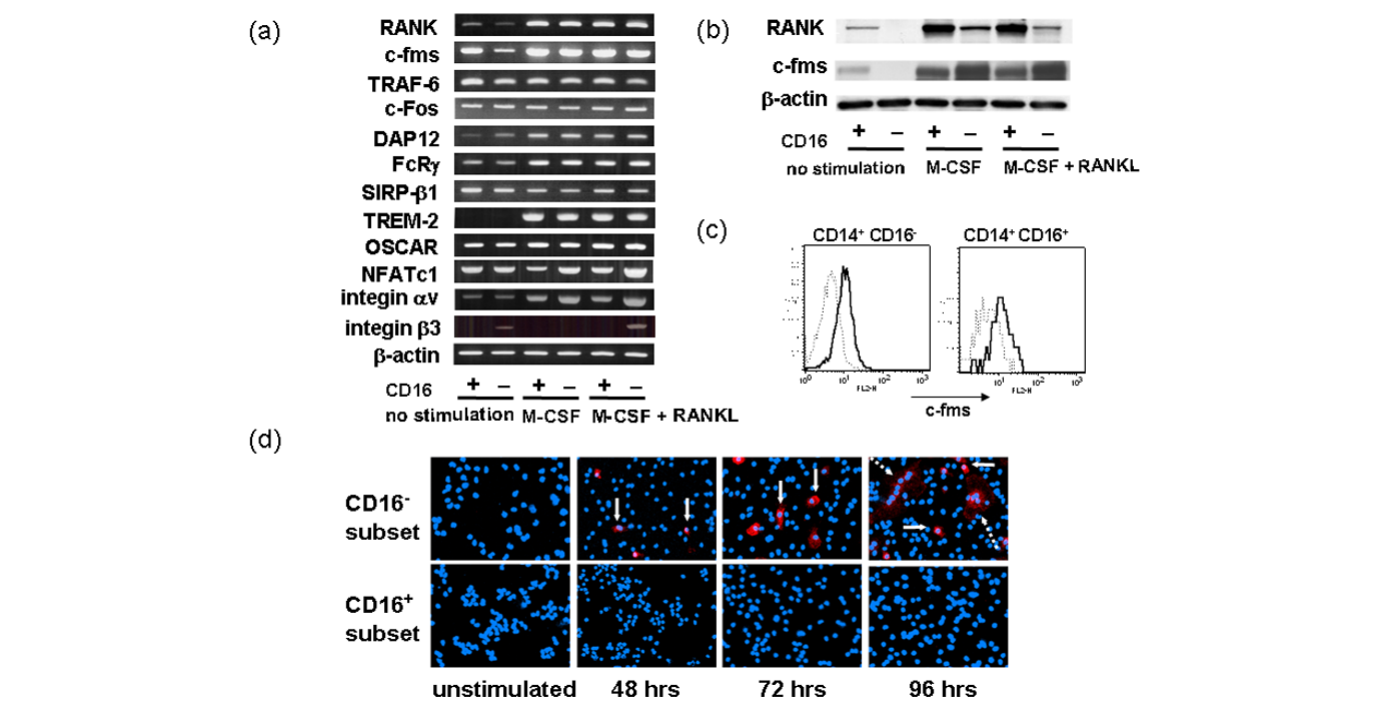
Differences in expression pattern of molecules related to osteoclastogenesis between CD16^+ ^and CD16^- ^monocyte subsets. (a) Total RNA was extracted from freshly isolated CD16^+ ^and CD16^- ^monocytes or from the cells incubated in either macrophage colony-stimulating factor (M-CSF) (25 ng/ml) alone or M-CSF (25 ng/ml) + receptor activator of NF-κB ligand (RANKL) (40 ng/ml) for 3 days, and semiquantitative RT-PCR analysis was performed. Representative results from three independent experiments are shown. (b) The expression of receptor activator of NF-κB (RANK) and c-fms in unstimulated or stimulated monocytes was analyzed by western blotting. (c) Cell surface expression of c-fms on unstimulated CD16^+ ^and CD16^- ^monocytes was examined by three-color flow cytometry. Gates were set either for CD14^+ ^CD16^+ ^(left panel) or CD14^+ ^CD16^+ ^(right panel) monocytes. Histograms show the stained cells with anti-c-fms mAb (solid lines) and isotype-matched control (dotted lines). (d) Purified monocytes were allowed to adhere on plates overnight (unstimulated) or the cells treated with M-CSF (25 ng/ml) + RANKL (40 ng/ml) were examined for the expression of the αvβ3 heterodimer by immunofluorescent staining. Solid arrows indicate mononuclear αvβ3-positive cells. Dotted arrows indicate multinucleated αvβ3-positive cells. Original magnification, ×100. Results are representative of two independent experiments.

The mRNA levels of RANK, c-fms, TRAF-6, DAP12 and SIRP-β1 under the baseline condition (no stimulation) varied between the donors; however, we did not find consistent differences in the mRNA levels of these molecules between the CD16^+ ^monocyte subset and the CD16^- ^monocyte subset among three to six donors (Figure 4a). The mRNA levels of other molecules, apart from integrin β3, were similar between the two subsets under the no-stimulation condition. Although the mRNA levels of RANK, c-fms, DAP12, FcRγ, TREM-2 and OSCAR increased in response to M-CSF alone or M-CSF + RANKL in both subsets, the expression levels were not significantly different between the two subsets. Expressions of TRAF-6, c-Fos and SIRP-β1 mRNA did not change following stimulation with M-CSF + RANKL. Of note, the expression of NFATc1 mRNA was enhanced by M-CSF + RANKL treatment only in the CD16^- ^subset. Furthermore, expression of integrin αv in both subsets was enhanced by M-CSF with or without RANKL; however, the expression level was greater in the CD16^- ^subset. It was noted that integrin-β3 mRNA was detected only in the CD16^- ^subset and was increased by M-CSF + RANKL stimulation, but not by M-CSF alone. The protein expression of RANK under the baseline condition was weakly detected in both subsets, and the levels were varied between donors by western immunoblotting.

The protein expression of c-fms was weakly detected in unstimulated CD16^+ ^monocytes, but not in CD16^- ^monocytes (Figure 4b). Flow cytometry analysis of c-fms in fresh monocytes, however, showed that both subsets express the molecule on the cell surface (Figure 4c). Expressions of both RANK and c-fms were upregulated by M-CSF alone and by M-CSF + RANKL, and we did not find consistent differences in the protein levels of these molecules between the two monocyte subsets. The profiles of expression levels of molecules involved in RANKL/RANK and its costimulatory pathways are similar between the two subsets, except for NFATc1, integrin αv and integrin β3. We therefore assumed that the distinct induction of NFATc1, integrin αv and integrin β3 in response to RANKL stimulation among the two monocyte subsets might explain the differences in their abilities to differentiate into osteoclasts.

### RANKL stimulation induces αvβ3 expression on CD16^- ^monocytes

The integrin-β3 subunit binds to integrin αv only and is expressed as the heterodimeric protein αvβ3 on monocytes and osteoclasts [17]. We examined the expression of αvβ3 on CD16^+ ^and CD16^- ^monocytes by immunofluorescent staining. Neither unstimulated nor M-CSF-stimulated monocyte subsets expressed αvβ3 (Figure 4d and data not shown). After 48 and 72 hours of treatment with M-CSF + RANKL, αvβ3-positive mononuclear cells were observed in CD16^- ^monocyte cultures but not in CD16^+ ^monocyte cultures. At 96 hours, both αvβ3-positive mononuclear cells and multinucleated cells were present in the CD16^- ^monocyte culture. The results indicated that αvβ3 was selectively expressed on CD16^- ^monocytes in the presence of M-CSF + RANKL, and the expression was revealed before the cells differentiate into typical multinucleated osteoclasts.

### RANKL activates ERK and p38 kinases only in CD16^- ^monocytes

**Figure 5:**
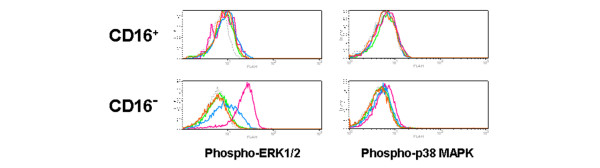
Flow cytometric analysis of ERK1/2 and p38 MAPK phosphorylation on monocyte subsets. Purified monocytes were precultured with macrophage colony-stimulating factor (M-CSF) for 3 days, and treated with 40 ng/ml receptor activator of NF-κB ligand (RANKL) for 5 min (pink), 10 min (blue) or 20 min (orange), or were left untreated (light green). The cells were then stained either with phospho-ERK1/2 (T202/Y204) or phospho-p38 MAPK (T180/Y182) after fixation and permeabilization. Isotype controls were shown in dotted line. The data shown are representative of three independent experiments.

Since ERK and p38 MAPK are essential in RANKL-induced osteoclastogenesis [18-20], we next examined whether these kinases were activated differently in CD16^+ ^monocytes and in CD16^- ^monocytes. Purified monocytes were precultured with 25 ng/ml M-CSF for 3 days to enhance RANK expression, and were then treated with RANKL. The RANKL treatment induced phosphorylation of both ERK and p38 MAPK in CD16^- ^monocytes at 5 minutes postexposure, although the p38 MAPK phosphorylation was weak. Both phosphorylations declined to a basal level within 20 minutes (Figure 5). In contrast, ERK and p38 MAPK were not detectably phosphorylated in CD16^+ ^monocytes with RANKL.

### siRNA targeting integrin β3 inhibits osteoclastogenesis from CD16^- ^monocytes

**Figure 6:**
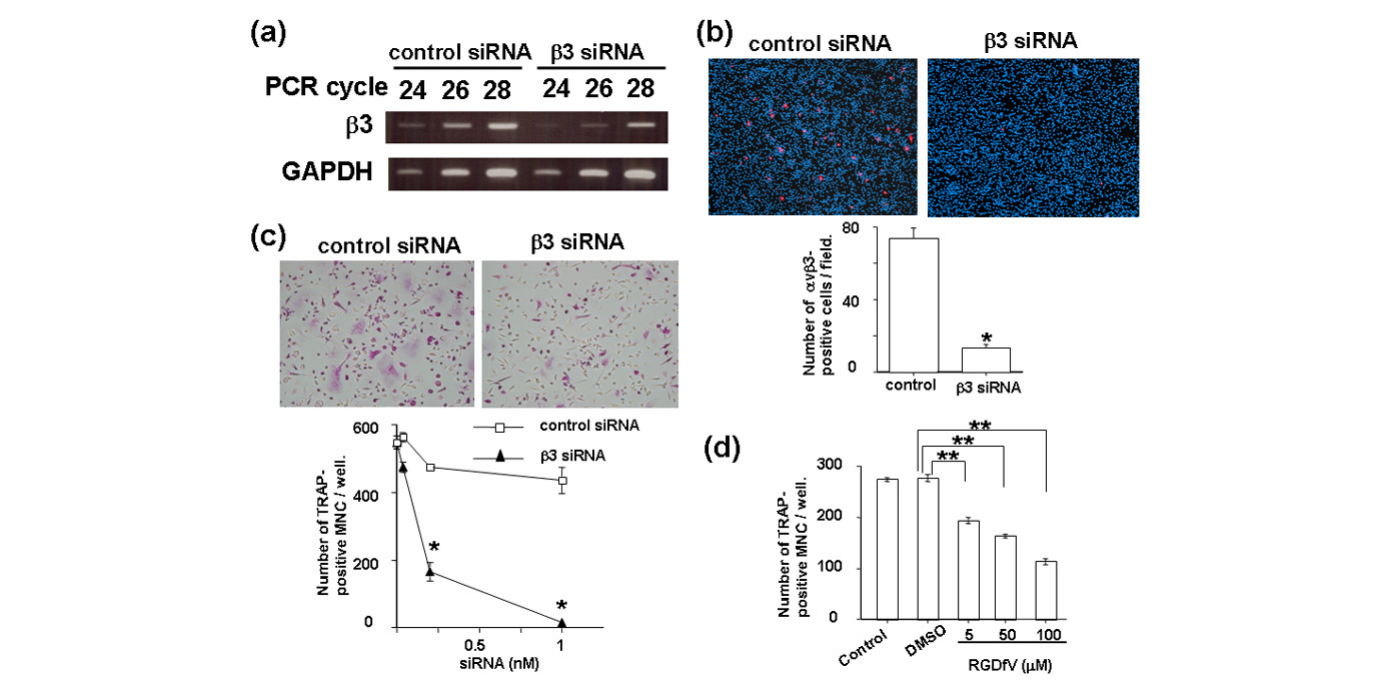
Effect of transfection of integrin-β3 siRNA and cyclic RGDfV peptide on osteoclastogenesis from CD16^- ^monocytes. (a) CD16^- ^monocytes transfected with either 1 nM control or integrin-β3 siRNA were cultured in macrophage colony-stimulating factor (M-CSF) (25 ng/ml) + receptor activator of NF-κB ligand (RANKL) (40 ng/ml). Forty-eight hours after the transfection, integrin-β3 mRNA levels were examined by semiquantitative RT-PCR. (b) The expression of the αvβ3 heterodimer was examined by immunostaining and the number of αvβ3-positive cells was counted. (c) Seven days after the transfection of siRNAs, the cells were stained for tartrate-resistant acid phosphatase (TRAP) activity, and the number of TRAP-positive multinucleated cells (MNC) was counted. Results are representative of three to five independent experiments. Data are the mean ± SEM values of duplicate wells. *P < 0.01, control-siRNA vs β3-siRNA. (d) CD16^- ^monocytes were incubated with M-CSF (25 ng/ml) + RANKL (40 ng/ml) for 2 days, followed by the addition of a medium containing either cyclic RGDfV peptide or dimethyl sulfoxide (DMSO). After incubation for a further 5 days, the number of TRAP-positive multinucleated cells (MNC) was counted. Representative results from three independent experiments are shown. Data are the mean ± standard error of the mean values of triplicate wells. Control, without treatment. **P < 0.03, DMSO vs cyclic RGDfV peptide.

The integrin-β3 cytoplasmic domain is essential for activation of intracellular signals from αvβ3 heterodimers [17]. We therefore examined the involvement of αvβ3 in RANKL + M-CSF-induced osteoclastogenesis in human CD16^- ^monocytes using siRNA targeting the integrin-β3 subunit. The integrin-β3 siRNA or control randomized siRNA were transfected into CD16^- ^monocytes. At 48 hours post-transfection, we determined the integrin-β3 mRNA level and αvβ3 heterodimer protein expression. The integrin-β3 mRNA level was reduced in the integrin-β3 siRNA-transfected monocytes compared with control siRNA-transfected monocytes (Figure 6a). The αvβ3 heterodimer expression was evaluated by immunofluorescent staining. The number of αvβ3-positive cells was significantly decreased in integrin-β3 siRNA-transfected monocytes compared with that in control siRNA (Figure 6b).

After 7 days of incubation, the number of TRAP-positive MNC was counted. Transfection with integrin-β3 siRNA significantly reduced the number of TRAP-positive MNC in a dose-dependent manner compared with control siRNA transfection (Figure 6c). In addition, the use of siRNA directed toward a different site of integrin-β3 mRNA also inhibited osteoclast formation from CD16^- ^monocytes (data not shown). On the other hand, siRNA that targeted lamin, which was used as a negative control, did not inhibit the induction of osteoclasts (data not shown). These results indicate the importance of integrin β3 in RANKL-induced osteoclast formation from CD16^- ^peripheral blood monocytes.

### Cyclic RGDfV peptide inhibits the osteoclastogenesis from CD16^- ^monocytes

Integrin αvβ3 recognizes a common tripeptide sequence, RGD (Arg-Gly-Asp), which is present in bone matrix proteins such as vitronectin and fibronectin [21]. Cyclic RGDfV peptide (Arg-Gly-Asp-D-Phe-Val) inhibits binding of the RGD-containing molecules to αvβ3 [22]. To investigate the role of ligand binding to the αvβ3 heterodimer in the osteoclastogenesis, we examined whether cyclic RGDfV peptide inhibits the formation of osteoclasts. Cyclic RGDfV peptide significantly reduced the number of TRAP-positive MNC in a dose-dependent manner (Figure 6d). The results imply possible involvement of ligand bindings to αvβ3 in the osteoclastogenesis.

### Knockdown of integrin β3 did not affect the expression of NFATc1 mRNA

**Figure 7:**
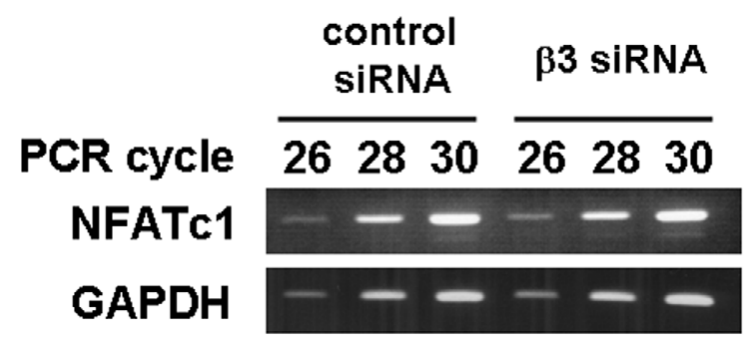
Effect of integrin-β3 knockdown on induction of NFATc1 mRNA. CD16^- ^monocytes transfected with either control or integrin-β3 siRNA were cultured with macrophage colony-stimulating factor (M-CSF) (25 ng/ml) + receptor activator of NF-κB ligand (RANKL) (40 ng/ml). Total RNA was extracted 48 hours post-transfection. Semiquantitative RT-PCR analysis was performed using NFATc1-specific and GAPDH-specific primers. Representative results from four independent experiments are shown.

In the next step, we determined whether integrin-β3-siRNA-induced inhibition of the osteoclastogenesis reflects downregulation of NFATc1, which is a key transcription factor in osteoclastogenesis [23]. For this purpose, we compared NFATc1 mRNA levels between integrin β3 and control siRNA-transfected monocytes. Interestingly, integrin-β3 knockdown did not alter the NFATc1 mRNA level (Figure 7), suggesting that signal transduction mediated by integrin β3 does not affect the expression of NFATc1.

### Detection of CD16^+ ^and CD16^- ^macrophages in synovium of RA patients

**Figure 8:**
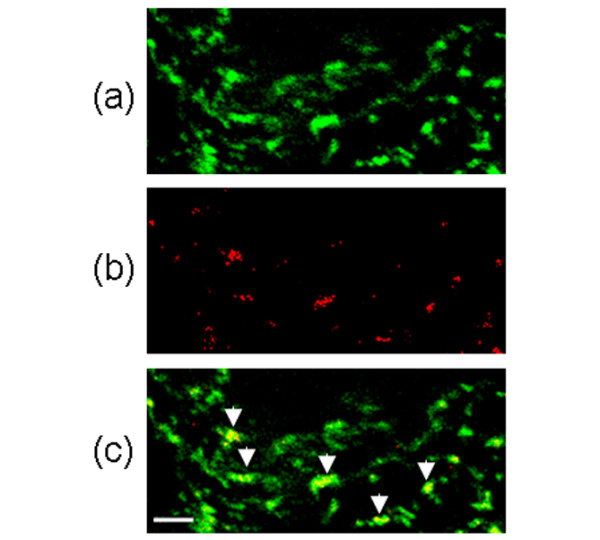
Double immunofluorescence showing CD16^+ ^and CD16^- ^macrophages in rheumatoid arthritis synovium. Synovial tissue samples from patients with rheumatoid arthritis (RA) were stained with CD68 and CD16. (a) CD68, (b) CD16, and (c) merged (a) with (b). Arrows show CD16^+ ^cells. Original magnification, ×400. Representative results from four RA patients are shown. Scale bar = 50 μm.

RA synovial macrophages are derived from peripheral blood monocytes, and their recruitment into the synovium is facilitated by various adhesion molecules and chemokines [24]. To analyze CD16 expression on synovial macrophages, RA synovial tissues were double-stained for CD16 and a macrophage marker, CD68. CD16^-^/CD68^+ ^macrophages were widespread in the synovium. Although less frequent, CD16^+^/CD68^+ ^macrophages were also observed both in the synovial intima and subintima (Figure 8). The presence of two subsets of macrophages, CD16^+ ^and CD16^-^, in RA synovium indicates that both CD16^+ ^and CD16^- ^peripheral blood monocytes are recruited into the synovium.

## Discussion

Human peripheral blood monocytes are a heterogeneous population, and they are divided into two subsets based on the expression of CD16. The CD16^+ ^and CD16^- ^monocyte subsets show functional differences in migration, cytokine production and differentiation into macrophages or dendritic cells [11-13,15]. We focused on the heterogeneity of the monocytes, and the primary question addressed in this study was which monocyte subset could be the source of osteoclasts. The results demonstrated that CD16^- ^peripheral blood monocytes, but not CD16^+ ^monocytes, differentiated *in vitro *into osteoclasts by treatment with RANKL + M-CSF.

To investigate the molecular mechanisms of the different response to RANKL and the differentiation into osteoclasts between CD16^+ ^and CD16^- ^monocytes, we examined the expression of molecules known to be involved in osteoclastogenesis. The expression profiles of integrin αv, integrin β3 and NFATc1 were different between the two subsets. Integrin αvβ3 heterodimer was expressed only on RANKL and M-CSF-stimulated CD16^- ^monocytes. It is known that αvβ3 expressed on osteoclasts is important in bone resorption as well as in attachment of osteoclasts to the bone matrix [25].

It was recently reported that bone marrow macrophages of integrin-β3-deficient mice could not differentiate into mature osteoclasts *in vitro*, suggesting that αvβ3 is involved not only in activation, but also in differentiation, of osteoclasts in mice [26,27]. The authors also showed that αvβ3 and c-fms share a common intracellular signaling pathway, including the activation of ERK and the induction of c-Fos [27], both of which are essential for osteoclastogenesis [28,29]. In addition, it was reported that echistatin, an αvβ3 antagonist, inhibited osteoclast formation of mouse bone marrow cells [30].

In accordance with these reports, our data showed that knockdown of integrin-β3 expression resulted in downregulation of the αvβ3 heterodimer, and abrogated osteoclastogenesis from human peripheral blood CD16^- ^monocytes. We also showed that blocking of adhesive ligands to bind to αvβ3 by RGDfV peptide inhibited osteoclast formation from CD16^- ^monocytes. Taken together, the process of ligand binding to αvβ3 may be involved in the osteoclastogenesis. Blockade of αvβ3 could therefore be a therapeutically beneficial approach to modulate osteoclastogenesis. Indeed, integrin αvβ3 antagonists effectively treated osteoporosis in mice, rats and humans, and protected bone destruction in rat adjuvant-induced arthritis *in vivo *[31-34]. Of note, it is reported that patients with Iraqi-Jewish-type Glanzmann thrombasthenia who are deficient in integrin β3 do not develop osteopetrosis because of the upregulation of α2β1 expression on osteoclasts, although the bone-resorptive ability of the osteoclasts was decreased *in vitro *[35]. The function of αvβ3 *in vivo *in osteoclast formation and resorptive function could therefore be partially compensated by other integrins.

Although all the multinucleated osteoclasts expressed αvβ3 (Figure 4d) [36], a small number of M-CSF + RANKL-stimulated mononuclear CD16^- ^monocytes expressed αvβ3 (Figure 4d). Multinucleated osteoclasts are formed by fusion of osteoclast precursor cells [37]. It was reported that αvβ3 is involved in the migration of osteoclast precursors [30]. The αvβ3-positive cells could therefore be forced to migrate by the ligands and may fuse with closed αvβ3-negative cells. Alternatively, only αvβ3-positive cells may be fused with each other.

It is possible to consider that signaling from CD16 by anti-CD16 mAb-coated magnetic beads, which were used for the cell separation, or by IgG contained in FBS might inhibit osteoclastogenesis from CD16^+ ^monocytes. We therefore separated the two subsets using anti-CD33 mAb and a fluorescent cell sorter, and stimulated the cells with M-CSF + RANKL. The results showed that CD33^low ^monocytes, which correspond to CD16^+ ^monocytes, still could not differentiate into osteoclasts. CD16 is a heterodimer consisting of FcγIIIa and Fcγ, and has low affinity for the Fc region of IgG. Aggregation of CD16 by immune complexes leads to transmission of activating signals via the immunoreceptor tyrosine-based activation motif in the γ chain [38]. We also assessed osteoclastogenesis from the two monocyte subsets using IgG-depleted bovine serum. Even in the IgG-free medium, CD16^- ^monocytes but not CD16^+ ^monocytes differentiated into osteoclasts (data not shown). We could therefore exclude the possibility that signal transduction through CD16 inhibits osteoclastogenesis from CD16^+ ^monocytes.

NFATc1 is a key transcription factor in osteoclastogenesis [16]. In the present study, stimulation with M-CSF + RANKL increased the NFATc1 mRNA expression in the CD16^- ^subset only, similar to integrin αv and integrin β3. The differences in NFATc1 induction might therefore also explain the difference in osteoclastogenesis between the two monocyte subsets. It is of interest that knockdown of integrin β3 did not lower the mRNA level of NFATc1. This result supports the notion that NFATc1 is located upstream of integrin-β3 expression [39]. It is also possible that parallel activation of two signaling pathways mediated by integrin β3 and NFATc1 contributes to osteoclastogenesis independently or cooperatively. Further studies are needed to determine the mechanisms of integrin β3 involvement in RANKL/RANK-mediated osteoclast differentiation.

It has been demonstrated that MAPK families, ERK and p38 MAPK, were activated by RANKL-induced intracellular signalings in osteoclasts and osteoclast precursors [18,19]. In addition, these kinases are involved in the differentiation of osteoclasts [20]. We showed that RANKL stimulation induced phosphorylation of ERK and p38 MAPK only in CD16^- ^monocytes. It is suggested that differential activation of these kinases may partially explain the distinct properties of the two monocyte subsets upon RANKL stimulation.

Our results showed that CD16^+ ^monocytes produce higher levels of inflammatory cytokines including TNFα and IL-6 compared with CD16^- ^monocytes. These results are consistent with the previous report showing that CD16^+ ^monocytes produced larger amounts of TNFα upon lipopolysaccharide or lipopeptide stimulation than did CD16^- ^monocytes [40]. Interestingly, we showed that stimulation either with RANKL or M-CSF upregulated the TNFα and IL-6 production by CD16^+ ^monocytes. A marked increase of CD16^+ ^monocytes in peripheral blood is reported in inflammatory diseases, such as infection, malignancy, Kawasaki disease and RA [41-44]. Taken together, CD16^+ ^monocytes may be an important source of inflammatory cytokines.

In mice, peripheral blood Ly-6C^high ^monocytes, which are thought to correspond to human CD16^- ^monocytes, increase in inflammatory conditions, and these cells are recruited into sites of inflammation [45]. In contrast, Ly-6C^low ^monocytes, which are thought to correspond to human CD16^+^, migrate into noninflamed tissues [12]. These data on mouse monocytes seem to be in contrast to the data on human monocytes, which show expansion of CD16^+ ^monocytes in inflammatory conditions where they produce larger amounts of inflammatory cytokines. At present, it is not clear whether mouse monocyte subsets, Ly-6C^low^/Ly-6C^high^, represent human monocyte subsets, CD16^+^/CD16^- ^monocytes, and whether the biologic functions of mouse monocytes are analogous to those of human monocytes.

In mice, blood monocytes newly released from the bone marrow are exclusively Ly-6C^high ^and the level of Ly-6C is downregulated while in circulation [45]. It is thus suggested that in mice the two monocyte subsets differing in Ly-6C expression represent different stages in the maturation pathway. In the human, transition from CD16^- ^monocytes to CD16^+ ^monocytes is observed upon culture with IL-10, M-CSF and transforming growth factor beta *in vitro *[42,46]. Similar to mouse monocytes, therefore, human peripheral blood CD16^- ^monocytes may also maturate into CD16^+ ^monocytes.

It is reported that a significant number of RA synovial cells in the intima express CD16, suggesting that CD16^+ ^cells are synovial macrophages [47]. We confirmed that both CD16^+ ^and CD16^- ^macrophages accumulate in the RA synovium by double-color immunohistochemical staining for CD68 and CD16. A number of chemokines are abundantly expressed in the RA synovium [24,48]. Among these cytokines, MCP-1, MIP-1α, SDF-1, RANTES and fractalkine can induce migration of CD16^- ^monocytes *in vitro *([11,12] and unpublished data). On the other hand, migration of CD16^+ ^monocytes is induced only by fractalkine. These chemokines therefore seem to play an important role in recruitment of CD16^+ ^and CD16^- ^monocytes from the circulating pool into the RA synovium.

The osteoclast inducers are also produced in the RA synovium. RANKL is expressed by synovial fibroblasts and activated T cells [49-51], while M-CSF is expressed on RA synovial macrophages and fibroblasts [52,53].

TNFα and IL-6, which are mainly expressed on RA synovial macrophages and fibroblasts, respectively, could also enhance osteoclast differentiation [54]. Collectively, it is probable that the recruited CD16^- ^monocytes/macrophages differentiate into osteoclasts in the RA synovium, and contribute to bone destruction. On the other hand, CD16^+ ^monocytes/macrophages might also be involved in RA pathogenesis by producing inflammatory cytokines including TNFα and IL-6. Since TNFα and IL-6 enhance osteoclast formation [54,55], CD16^+ ^monocytes/macrophages may also contribute to osteoclastogenesis in RA synovium.

## Conclusion

We have shown that human peripheral blood monocytes consist of two functionally heterogeneous subsets with distinct response to osteoclastogenic stimuli. Osteoclasts seem to originate from CD16^- ^monocytes, and integrin β3 is necessary for the osteoclastogenesis. The blockade of accumulation and activation of CD16^- ^monocytes could therefore be a beneficial approach as an anti-bone resorptive therapy, especially for RA.

## Abbreviations

DAP = DNAX-activation protein; ELISA = enzyme-linked immunosorbent assay; FBS = fetal bovine serum; FcRγ = Fc receptor γ chain; IL = interleukin; FITC = fluorescein isothiocianate; mAb, monoclonal antibody; M-CSF = macrophage colony-stimulating factor; MEM = modified Eagle's medium; MMP = matrix metalloproteinase; MNC = multinucleated cells; NF = nuclear factor; OSCAR = osteoclast-associated receptor; PBS = phosphate-buffered saline; PCR = polymerase chain reaction; RA = rheumatoid arthritis; RANK = receptor activator of NF-κB; RANKL = receptor activator of NF-κB ligand; RT = reverse transcriptase; siRNA = small interfering RNA; SIRP-β1 = signal regulatory protein-β1; TNFα = tumor necrosis factor alpha; TRAF = tumor necrosis factor receptor-associated factor; TRAP = tartrate-resistant acid phosphatase; TREM = triggering receptor expressed on myeloid cells.

## Competing interests

The authors declare that they have no competing interests.

## Authors' contributions

YK participated in the design of the study, carried out the experiments and statistical analysis, and drafted the manuscript. KH and KT participated in the design of the study and its coordination. TN and NM conceived of the study, participated in its design and coordination, and helped to draft the manuscript. All authors read and approved the final manuscript.

## Figures and Tables

**Table 1:** Primer sequences

Molecule	Primer sequence
Receptor activator of NF-κB	5'-TGG CCG CCT AAG TGG AGA TA
	3'-TGC GTA GGG ACC ACC TCC TA
c-fms	5'-GAC GTT TGA GCT CAC CCT TCG ATA
	3'-CCT GGT ACT TGG GCT TCT GCT TAT
Tumor necrosis factor receptor-associated factor 6	5'-AGA CAA GAC CAT CAA ATC CGG GAG
	3'-TCC AGG GCT ATG AAT CAC AAC AGG
c-Fos	5'-CAG GAG ACA GAC CAA CTA GA
	3'-TTC ACG GAC AGA TAA GGT CC
DNAX-activation protein 12	5'-ATG GGG GGA CTT GAA CCC
	3'-TCA TTT GTA ATA CGG CCT CTG TG
Fc receptor γ chain	5'-TGA TTC CAG CAG TGG TCT TGC TCT
	3'-ATG CAG GCA TAT GTG ATG CCA ACC
Signal regulatory protein β1	5'-ACC CAC CTT GGA GGT TAC TCA ACA
	3'-TGT AGA TGG CAG AGA CAC CAA CCA
Triggering receptor expressed on myeloid cells 2	5'-ATG GAG CCT CTC CGG GTG CT
	3'-CTG CGG AAT CTA CAA CCC CA
Osteoclast-associated receptor	5'-GAG TAG CTG AAA GGA AGA CGC GAT
	3'-CAG AGC GCT GAT TGG TCC ATC TTA
Nuclear factor of activated T cells c1	5'-TGT GCC GGA ATC CTG AAA CTC AGA
	3'-TCC CGT TGC AGA CGT AGA AAC TGA
Integrin αv	5'-TCC CAT CAG TGG TTT GGA GCA TCT
	3'-TCC GAC AGC CAC AGA ATA ACC CAA
Integrin β3	5'-TGC CTC AAC AAT GAG GTC ATC CCT
	3'-AGA CAC ATT GAC CAC AGA GGC ACT
β-Actin	5'-GTC CTC TCC CAA GTC CAC ACA
	3'-CTG GTC TCA AGT CAG TGT ACA GGT AA
GAPDH	5'-TGA TGA CAT CAA GAA GGT GGT GAA G
	3'-TCC TTG GAG GCC ATG TGG GCC AT
